# Supplementary material for: A PX-BAR protein Mvp1/SNX8 and a dynamin-like GTPase Vps1 drive endosomal recycling
Source: eLife. 2021 Sep 15;10:e69883. doi: 10.7554/eLife.69883 (PMC8504969; doi:10.7554/eLife.69883)
Supplement: Figure 1—source data 2. [file elife-69883-fig1-data2.docx]

Figure 1-source data 3.

The list of localization altered in retromer mutants. The list of endosomal transmembrane proteins whose examined their localization was examined in vps35Δ cells.

| Cargo | Localization was altered in *vps35*Δ cells |
| --- | --- |
| Vps10 | Yes |
| Pep12 | Yes |
| Ear1 | Yes |
| Mrl1 | Yes |
| Tvp38 | Yes |
| Syn8 | Yes |
| Neo1 | Yes |
| Nhx1 | No |
| Vps55 | No |
