# Supplementary figures and images for: A PX-BAR protein Mvp1/SNX8 and a dynamin-like GTPase Vps1 drive endosomal recycling
Source: eLife. 2021 Sep 15;10:e69883. doi: 10.7554/eLife.69883 (PMC8504969; doi:10.7554/eLife.69883)

Figure 2F

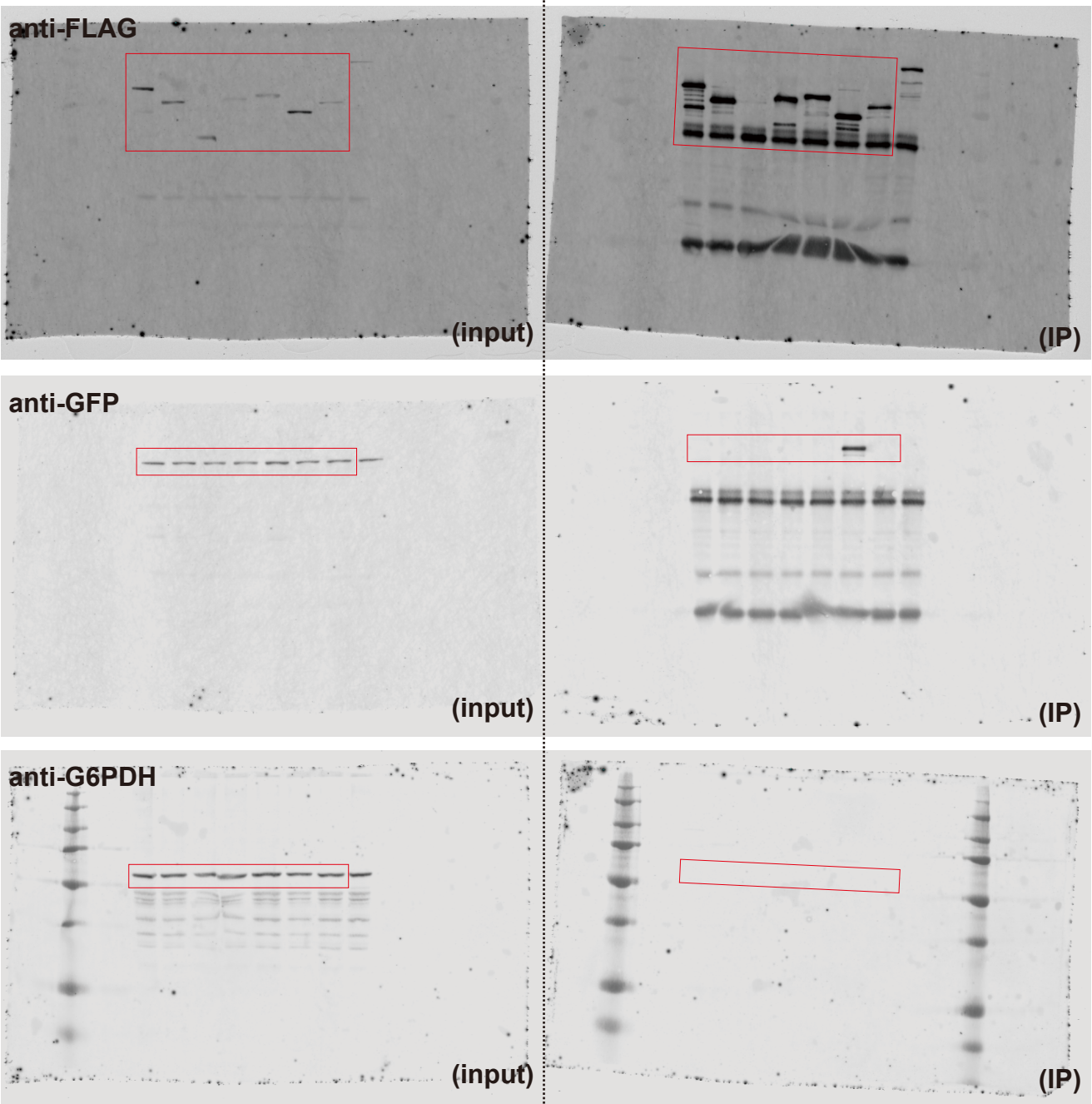

Supplement: Figure 2—source data 2. [file elife-69883-fig2-data2.pdf]

**Figure 2G**

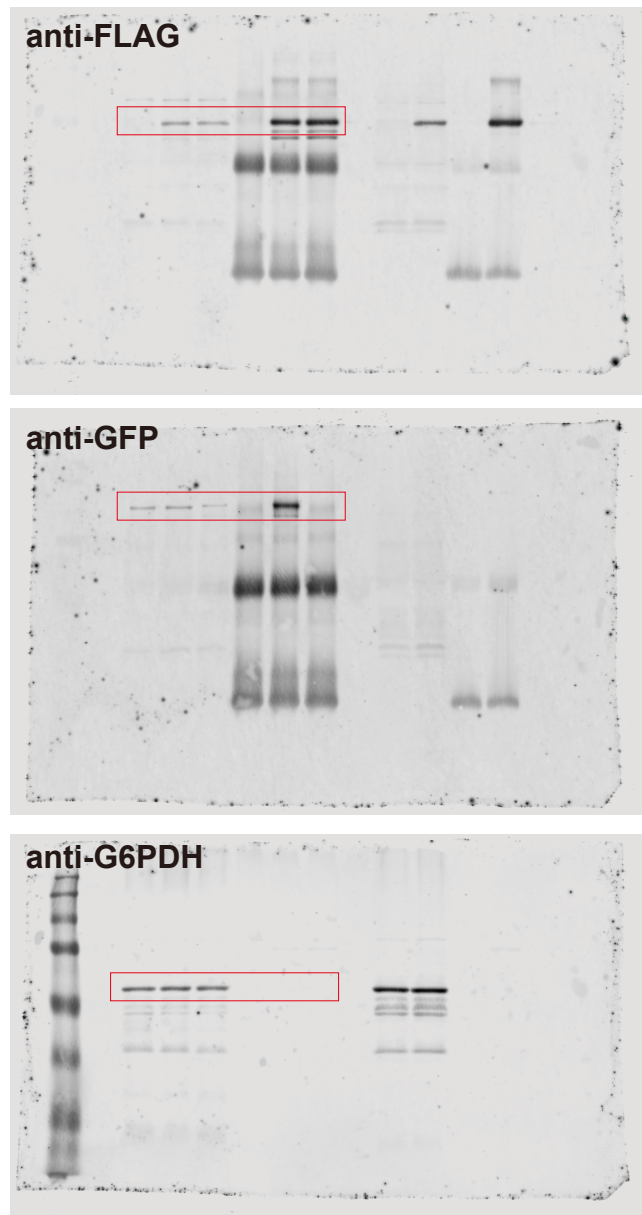

Supplement: Figure 2—source data 3. [file elife-69883-fig2-data3.pdf]

**Figure 3E**

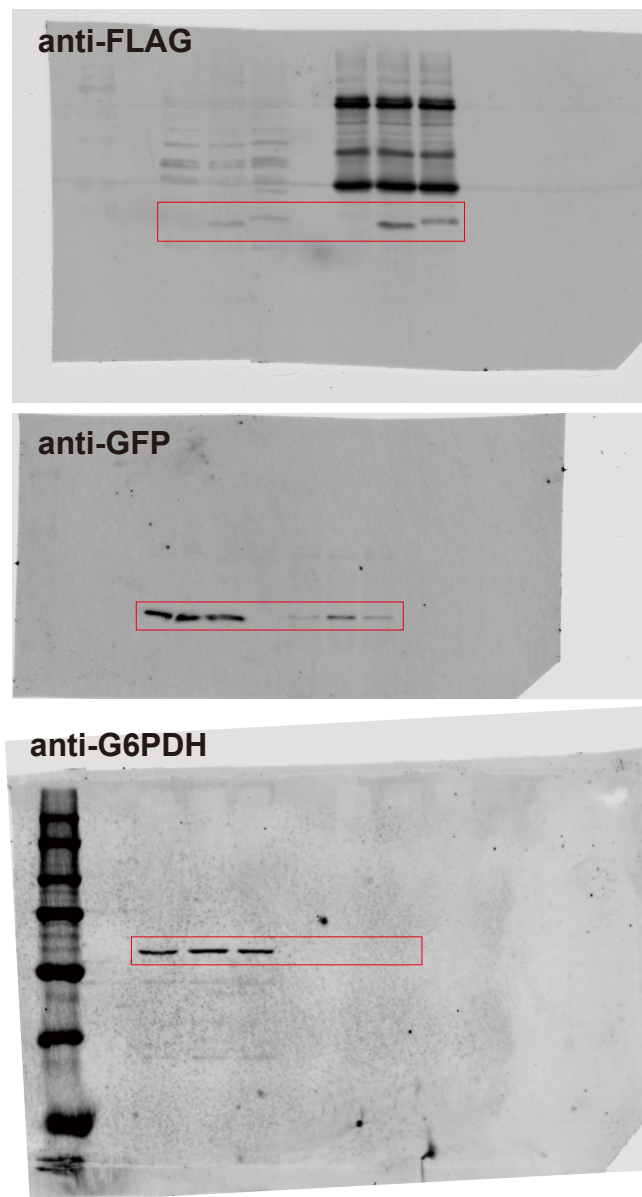

Supplement: Figure 3—source data 4. [file elife-69883-fig3-data4.pdf]

**Figure 3H**

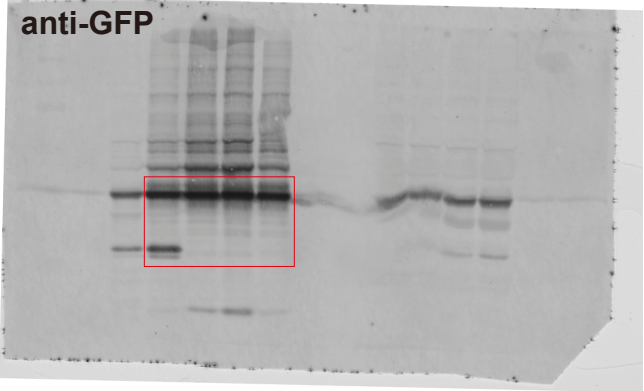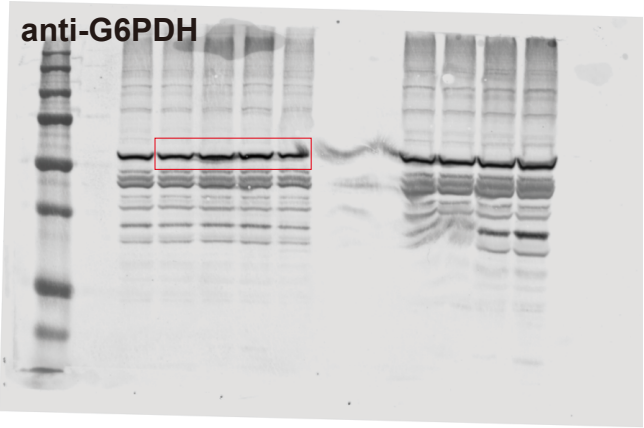

Supplement: Figure 3—source data 5. [file elife-69883-fig3-data5.pdf]

**Figure 3I**

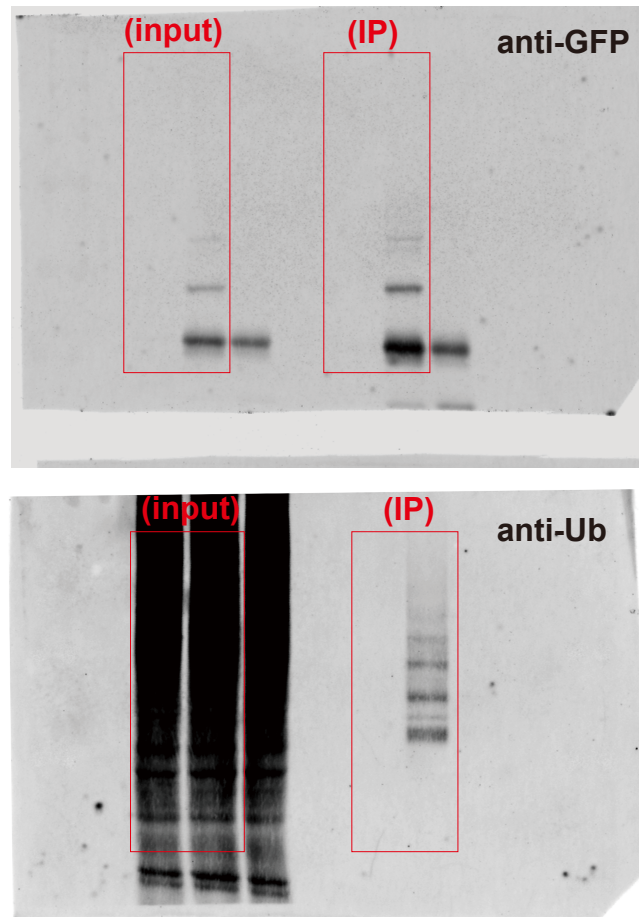

Supplement: Figure 3—source data 6. [file elife-69883-fig3-data6.pdf]

**Figure 3-figure supplement 1C**

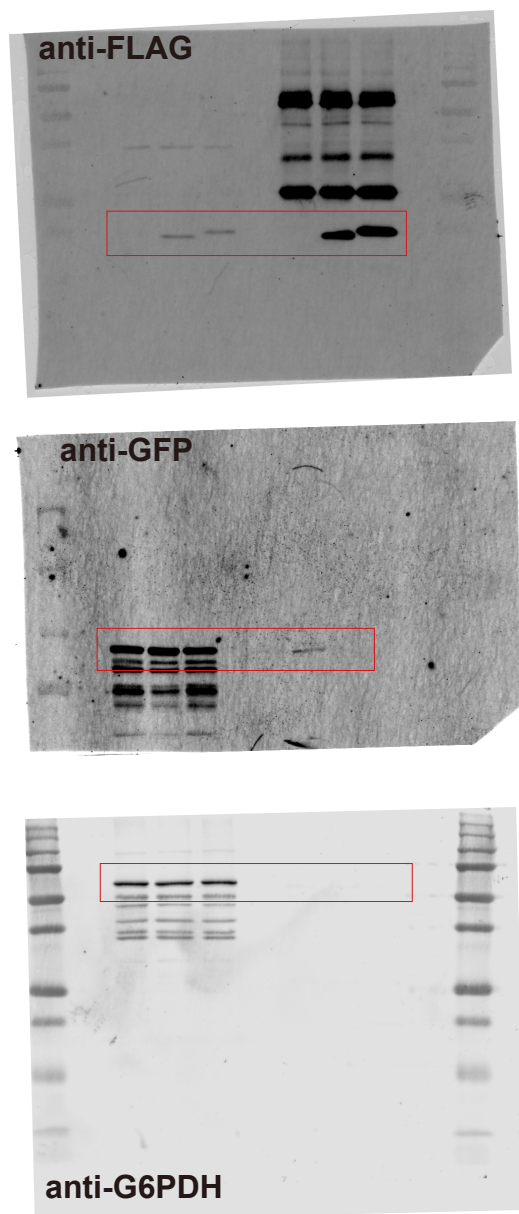

Supplement: Figure 3—figure supplement 1—source data 1. [file elife-69883-fig3-figsupp1-data1.pdf]

**Figure 3-figure supplement 1D**

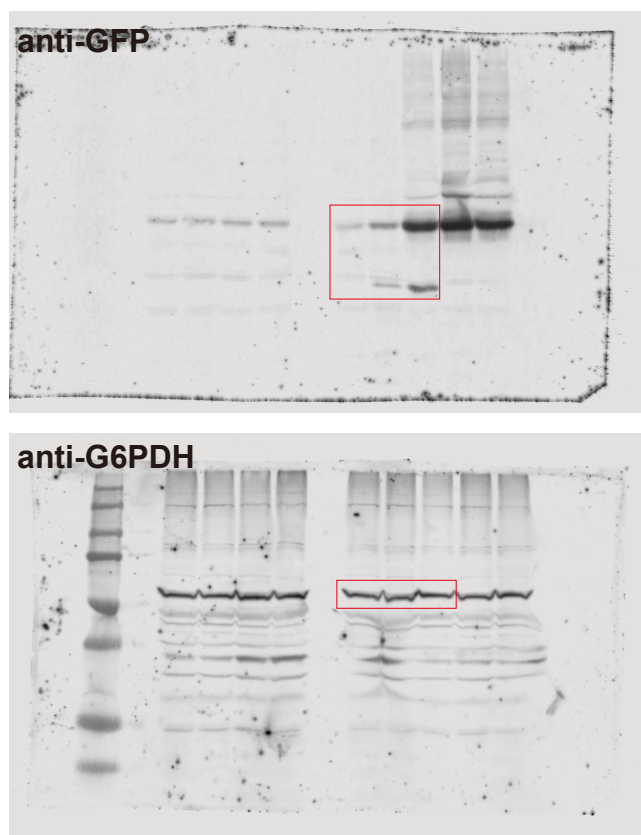

Supplement: Figure 3—figure supplement 1—source data 2. [file elife-69883-fig3-figsupp1-data2.pdf]

**Figure 3-figure supplement 1G**

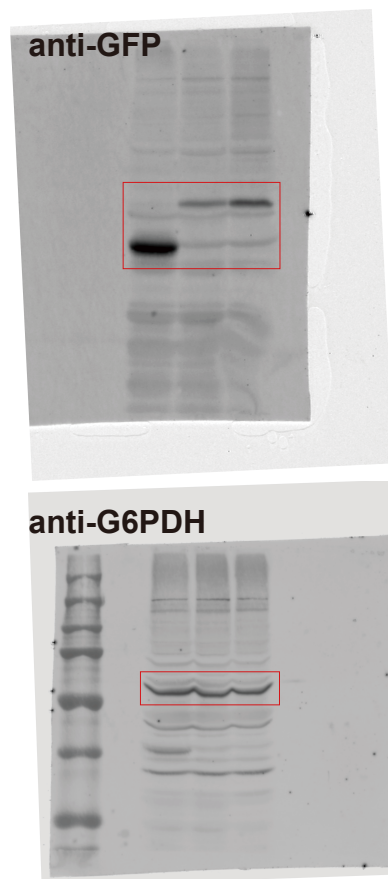

Supplement: Figure 3—figure supplement 1—source data 4. [file elife-69883-fig3-figsupp1-data4.pdf]

**Figure 4L**

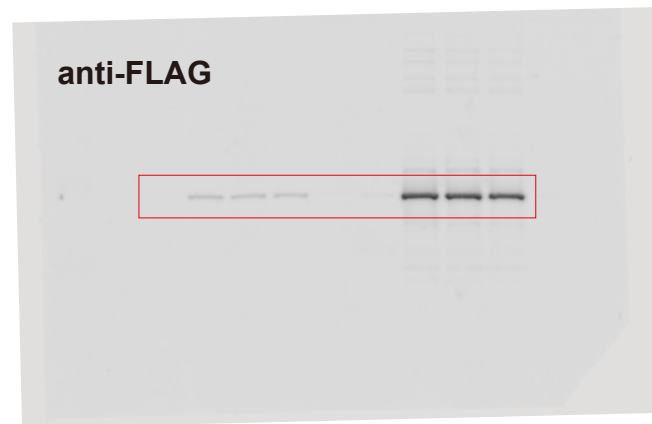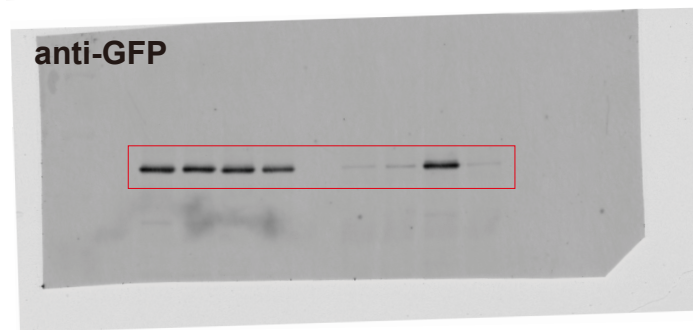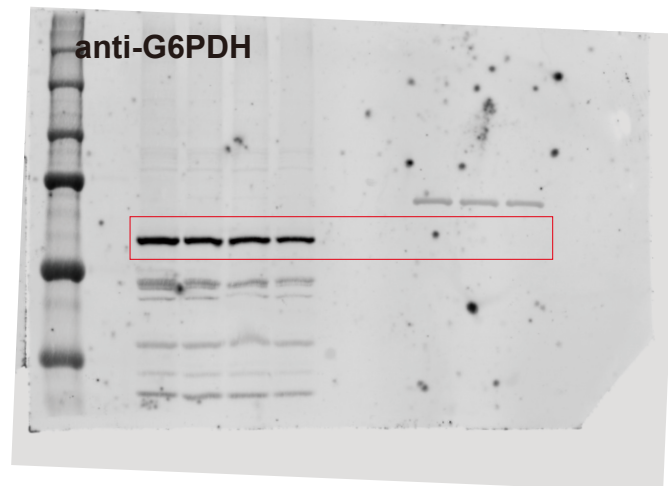

Supplement: Figure 4—source data 4. [file elife-69883-fig4-data4.pdf]

**Figure 4M**

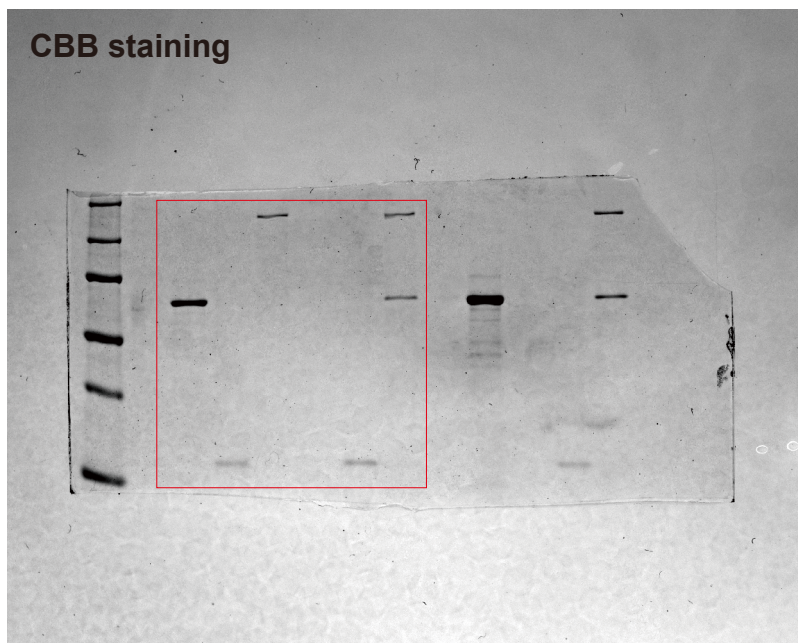

Supplement: Figure 4—source data 5. [file elife-69883-fig4-data5.pdf]

Figure 5D

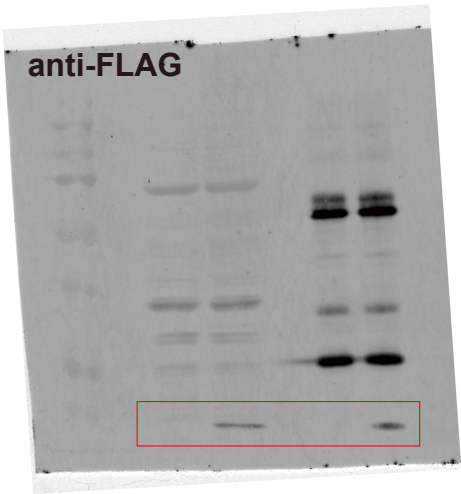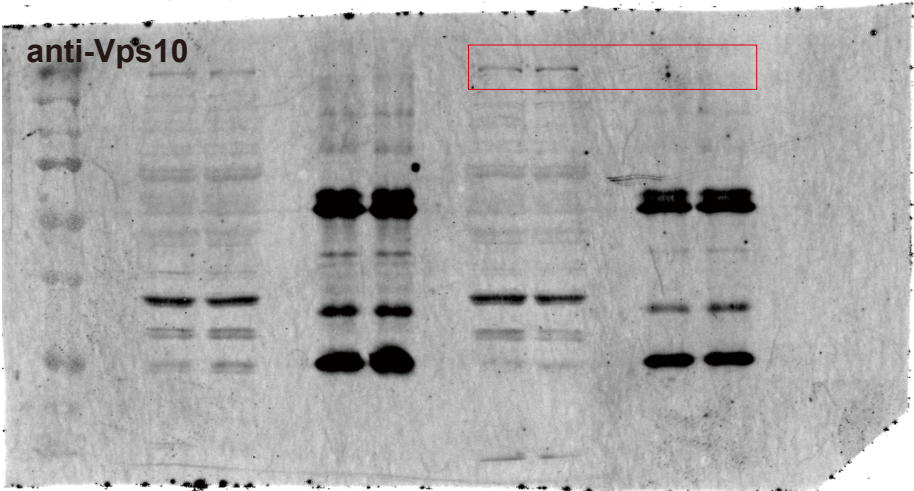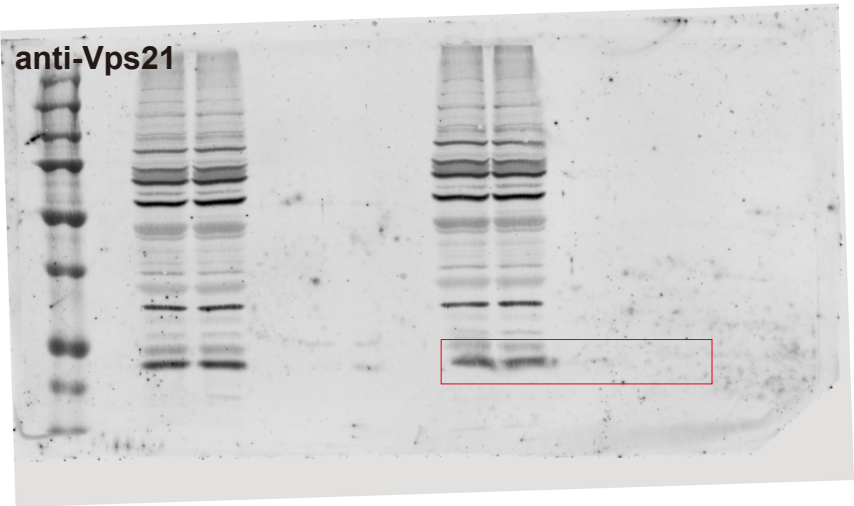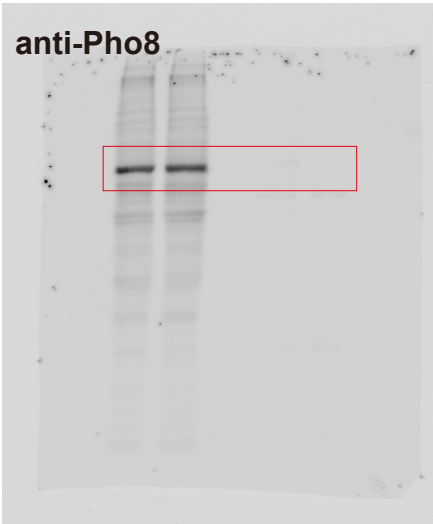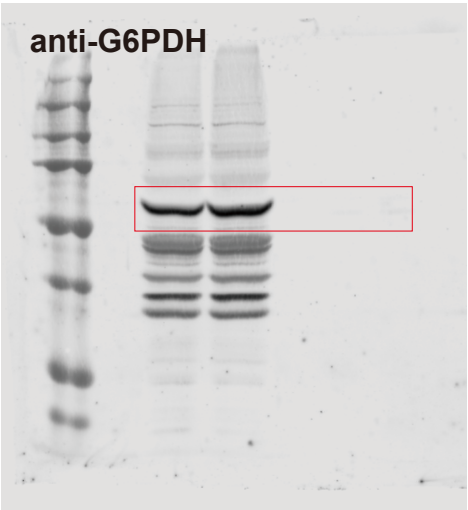

Supplement: Figure 5—source data 2. [file elife-69883-fig5-data2.pdf]

**Figure 5-supplemental 1A**

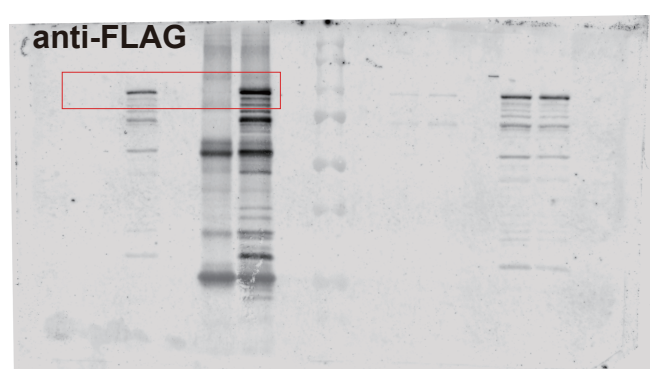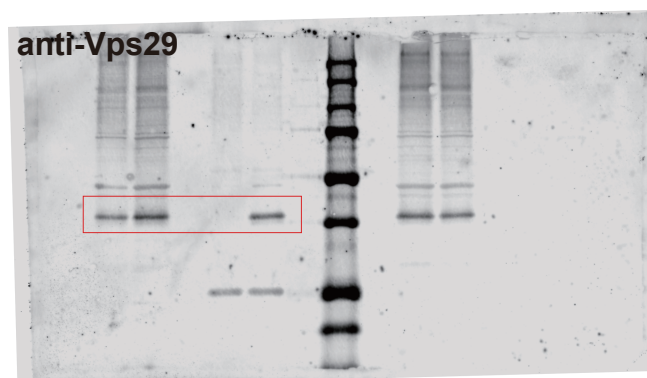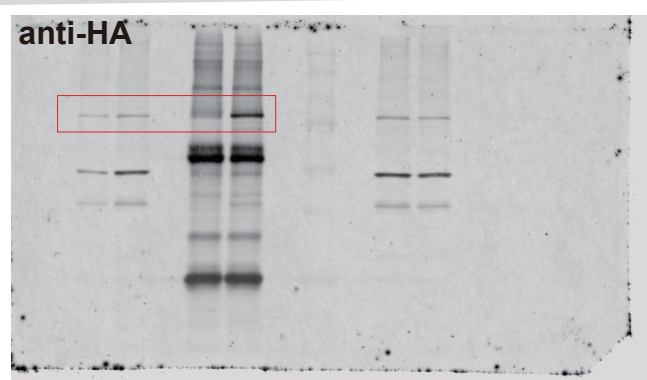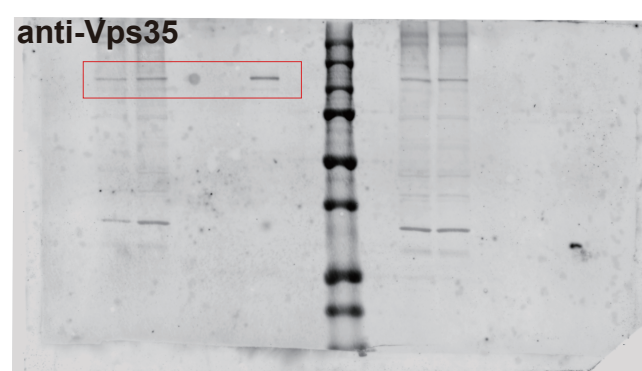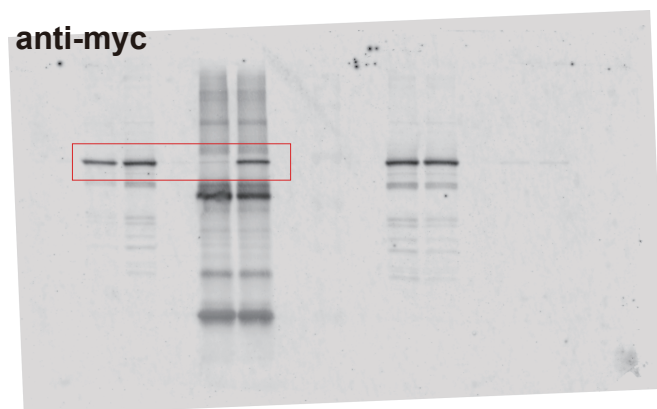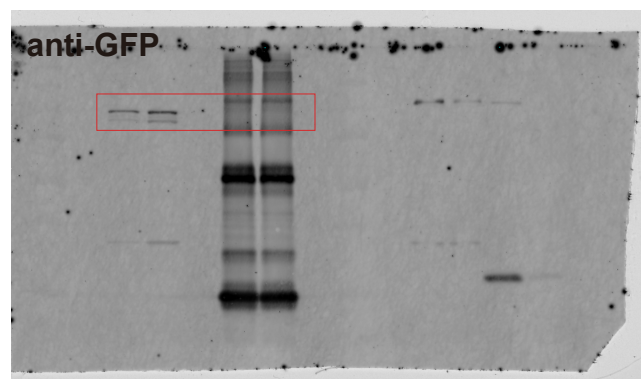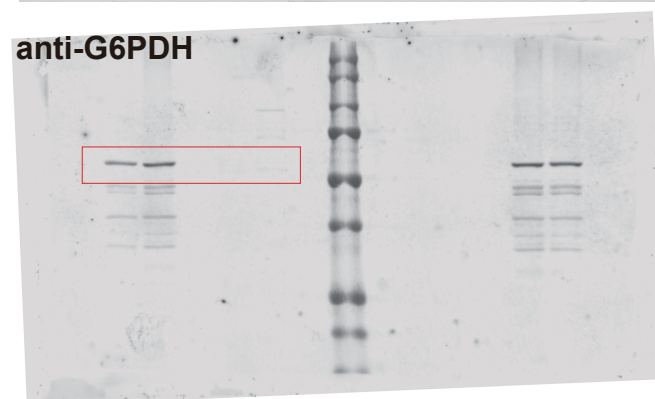

Supplement: Figure 5—figure supplement 1—source data 1. [file elife-69883-fig5-figsupp1-data1.pdf]

Figure 5-supplemental 1B

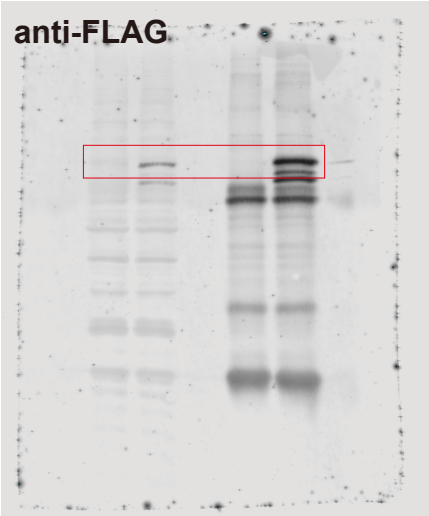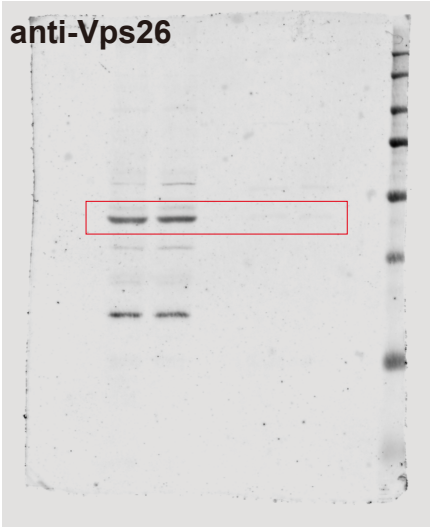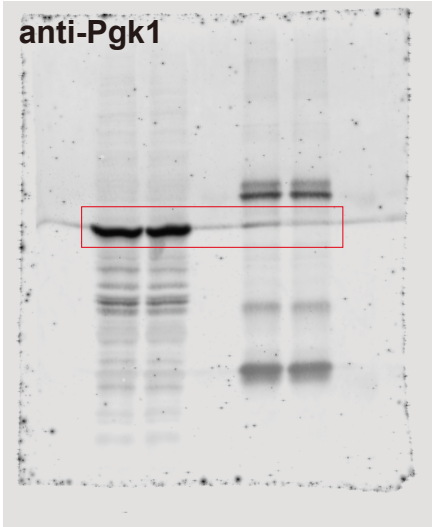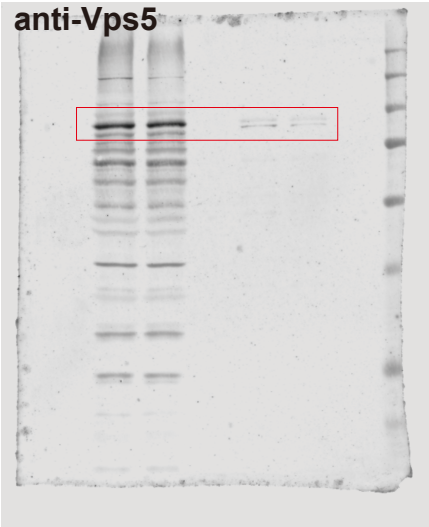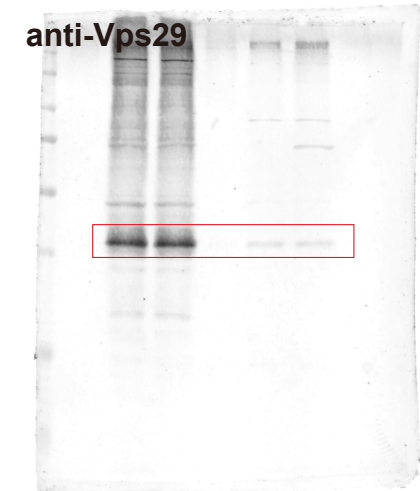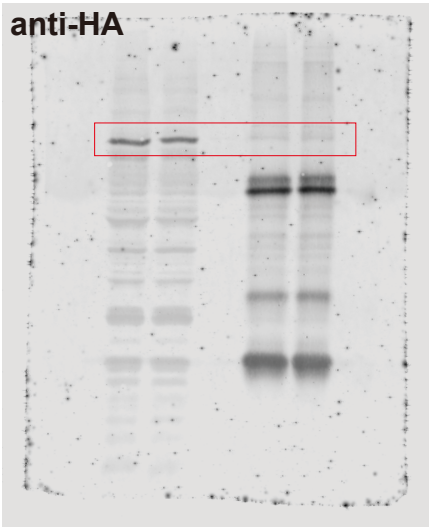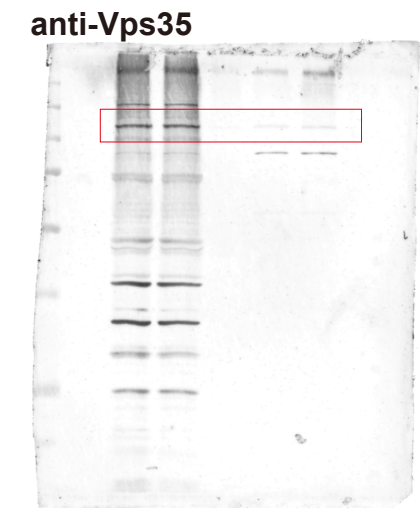

Supplement: Figure 5—figure supplement 1—source data 2. [file elife-69883-fig5-figsupp1-data2.pdf]

**Figure 5-supplemental 1J**

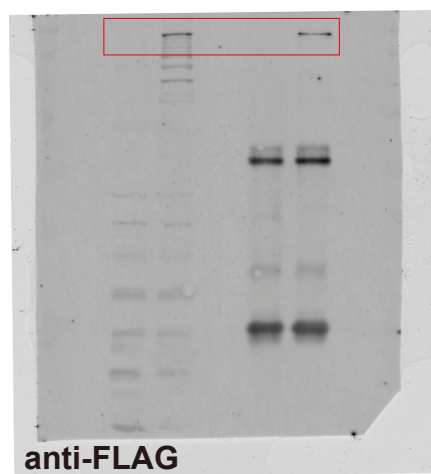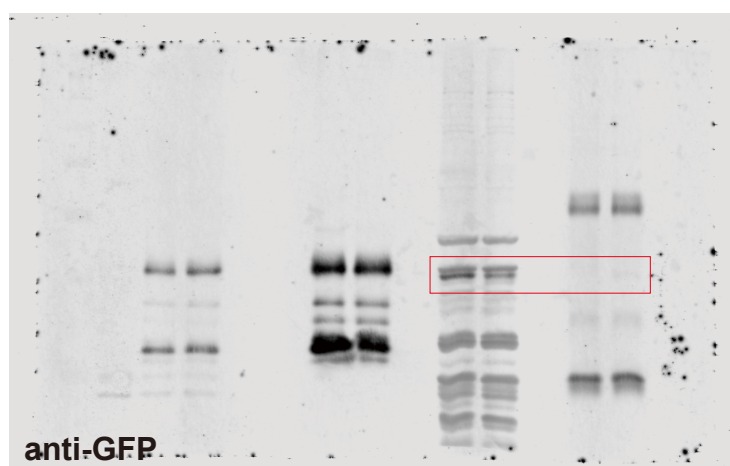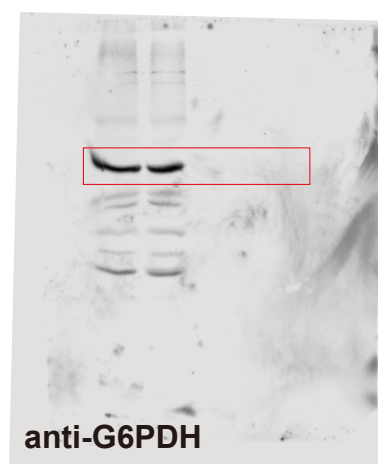

Supplement: Figure 5—figure supplement 1—source data 3. [file elife-69883-fig5-figsupp1-data3.pdf]

**Figure 6C**

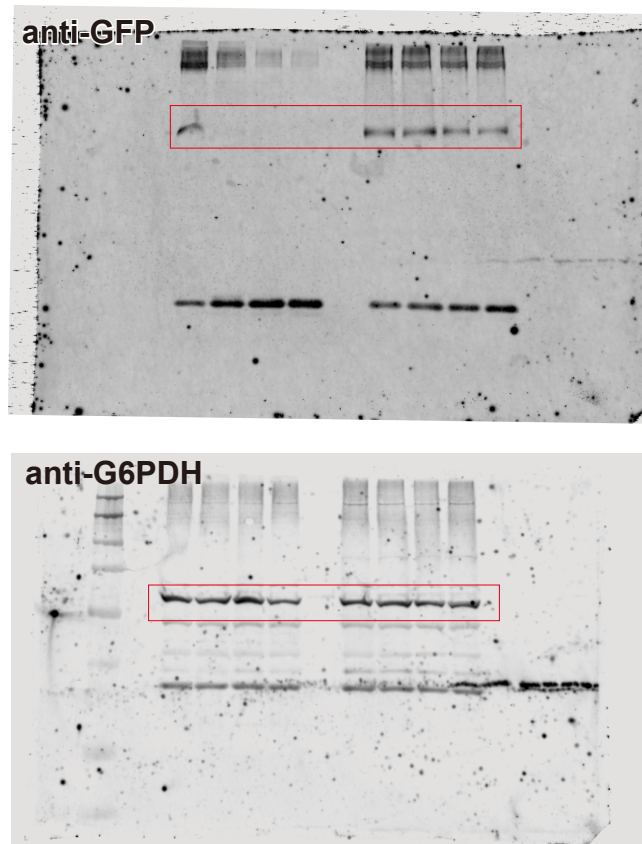

Supplement: Figure 6—source data 1. [file elife-69883-fig6-data1.pdf]

**Figure 6-supplemental 1A**

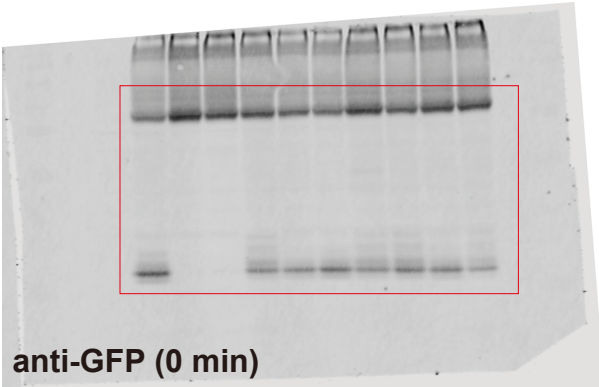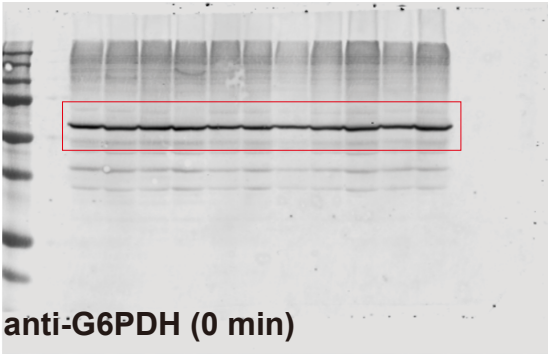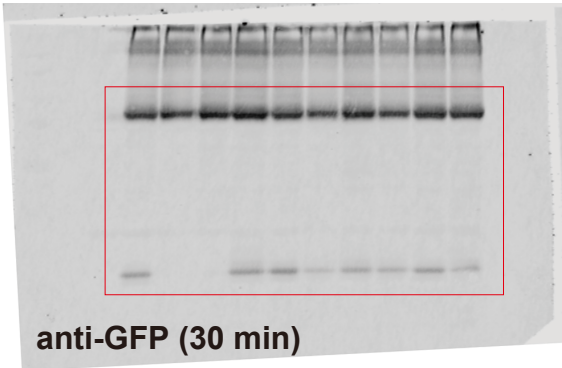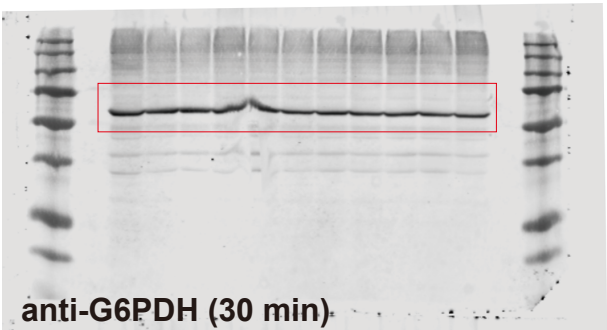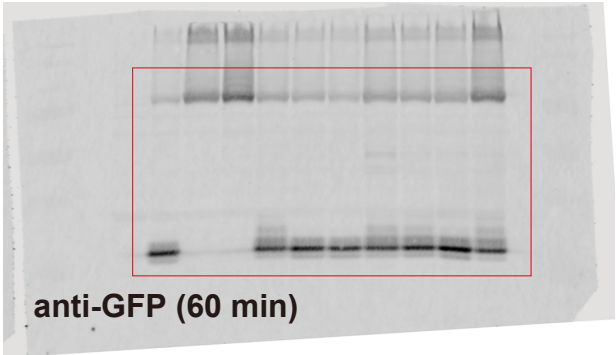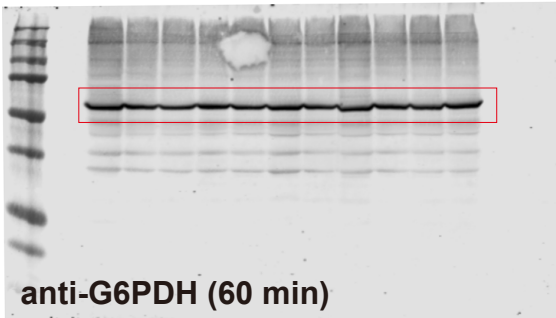

Supplement: Figure 6—figure supplement 1—source data 1. [file elife-69883-fig6-figsupp1-data1.pdf]
