# Supplementary material for: A PX-BAR protein Mvp1/SNX8 and a dynamin-like GTPase Vps1 drive endosomal recycling
Source: eLife. 2021 Sep 15;10:e69883. doi: 10.7554/eLife.69883 (PMC8504969; doi:10.7554/eLife.69883)
Supplement: Supplementary file 1. [file elife-69883-supp1.docx]

**Supplemental File 1A.** **Yeast strains used in this study**

| **Strain** | **Genotype** | **Source** |
| --- | --- | --- |
| SEY6210  SEY6210.1  SSY208  SSY209  SSY342  SSY413  SSY414  SSY778  SSY667  SSY252  SSY703  SSY659  SSY33  SSY970  SSY37  SSY615  SSY212  SSY490  SSY98  SSY1161  SSY259  SSY532  SSY83  SSY216  SSY1134  SSY479  SSY1128  SSY1129  SSY1130  SSY1131  SSY491  SSY474  SSY493  SSY477  SSY1240  SSY1271  SSY1232  SSY1178  SSY1241  SSY1180  SSY1244  SSY1068  SSY1069  SSY1272  SSY1070  SSY191  SSY1268  SSY1269  SSY1274  SSY1277  SSY1258  SSY1232  SSY1248  SSY727  SSY728  SSY439  SSY441  SSY445  SSY1171  SSY1169  SSY1264  SSY911  SSY913  SSY1032  SSY1033  SSY1034  SSY1035  SSY491  SSY1229  SSY792  SSY1015  SSY1217  SSY12118  SSY984  SSY985  SSY924  SSY1084  SSY1339  SSY1329  SSY1330 | *MAT*α *ura3-52 his3-200 leu2-3,112 trp1-901 lys2-801 suc2-9*  *MATa ura3-52 his3-200 leu2-3,112 trp1-901 lys2-801 suc2-9*  SEY6210, *vps35Δ::HIS3*  SEY6210, *snx4Δ::TRP1*  SEY6210, *mvp1Δ::TRP1*  SEY6210, *snx41Δ::TRP1*  SEY6210, *snx42Δ::TRP1*  SEY6210, *ykr078wΔ::TRP1*  SEY6210, *vps35Δ::KanMX6, snx4Δ::TRP1*  SEY6210, *vps35Δ::KanMX6, mvp1Δ:: TRP1*  SEY6210, *snx4Δ::KanMX6, mvp1Δ::TRP1*  SEY6210, *vps35Δ::KanMX6, snx4Δ::TRP1, mvp1Δ::hphNT1*  SEY6210, *pep4Δ::LEU2*  SEY6210, *pep4Δ::LEU2, prb1Δ::LEU2*  SEY6210, *vps4Δ::TRP1*  SEY6210, *vps34Δ::HIS3*  SEY6210, *pep12Δ::HIS3*  SEY6210, *vps55Δ::KanMX6*  SEY6210, *vps1Δ::KanMX6*  SEY6210, *VPS1-GFP::KanMX6*  SEY6210, *SEC7-mCherry::hphNT1*  SEY6210, *NHX1-2xmCherry::hphNT1*  SEY6210, *VPH1-mCherry::TRP1*  SEY6210, *VPH1-mCherry::TRP1*, *vps35Δ::KanMX6*  SEY6210, *VPH1-mCherry::TRP1*, *snx4Δ::KanMX6*  SEY6210, *VPH1-mCherry::TRP1*, *mvp1Δ::KanMX6*  SEY6210, *VPH1-mCherry::TRP1*, *vps35Δ::KanMX6, snx4Δ::TRP1*  SEY6210, *VPH1-mCherry::TRP1*, *vps35Δ::KanMX6, mvp1Δ::TRP1*  SEY6210, *VPH1-mCherry::TRP1*, *snx4Δ::TRP1, mvp1Δ::KanMX6*  SEY6210, *VPH1-mCherry::TRP1*, *vps35Δ::KanMX6, snx4Δ::TRP1, mvp1Δ::hphNT1*  SEY6210, *VPH1-mCherry::TRP1*, *vps55Δ::KanMX6*  SEY6210, *VPS55-GFP::LEU2*, *SEC7-mCherry::TRP1*  SEY6210, *VPS55-GFP::LEU2*, *mCherry-PEP12::URA3*  SEY6210, *MVP1-GFP::LEU2*, *mCherry-PEP12::URA3*  SEY6210, *VPS55-mNeonGreen-3xHA::KanMX6*, *mCherry-VPS21::LEU2*  SEY6210, *VPS55-mNeonGreen-3xHA::KanMX6*, *VPS10-mCherry::TRP1*  SEY6210, *MVP1-mNeonGreen-3xHA::KanMX6*, *mCherry-VPS21::LEU2*  SEY6210, *VPS1-GFP::HIS3*, *SEC7-mCherry::hphNT1*  SEY6210, *VPS1-GFP::KanMX6*, *mCherry-VPS21::LEU2*  SEY6210, *VPS1-GFP::HIS3*, *NHX1-2xmCherry::hphNT1*  SEY6210, *vps1Δ::hphNT1*, *MVP1-mRFP::URA3*  SEY6210, *VPS55-GFP::KanMX6*, *VPH1-mCherry::TRP1*  SEY6210, *VPS55-GFP::HIS3*, *VPH1-mCherry::TRP1*, *vps35Δ::KanMX6*  SEY6210, *VPS55-GFP::HIS3*, *VPH1-mCherry::TRP1*, *snx4Δ::KanMX6*  SEY6210, *VPS55-GFP::TRP1*, *VPH1-mCherry::TRP1*, *mvp1Δ::KanMX6*  SEY6210, *VPS55-GFP::TRP1*  SEY6210, *VPS55-GFP-Ub::HIS3*  SEY6210, *VPS55-GFP-Ub::HIS3, vps4Δ::TRP1*  SEY6210, *VPS55-mNeonGreen-3xHA::KanMX6*, *VPH1-mCherry::TRP1*  SEY6210, *VPS55-mNeonGreen-3xHA::KanMX6*, *VPH1-mCherry::TRP1*, *vps1Δ::hphNT1*  SEY6210, *VPS55-mNeonGreen-3xHA::KanMX6*, *mCherry-VPS21::LEU2*, *vps1Δ::hphNT1*  SEY6210, *MVP1-mNeonGreen-3xHA::KanMX6*, *mCherry-VPS21::LEU2*  SEY6210, *MVP1-mNeonGreen-3xHA::KanMX6*, *mCherry-VPS21::LEU2*, *vps1Δ::KanMX6*  SEY6210, *GFP-PHO8::URA3*  SEY6210, *GFP-PHO8::URA3*, *vps35Δ::KanMX6, snx4Δ::TRP1, mvp1Δ::hphNT1*  SEY6210, *GFP-SNC1::URA3*  SEY6210, *GFP-SNC1::URA3,* *snx4Δ::KanMX6*  SEY6210, *GFP-SNC1::URA3*, *mvp1Δ::TRP1*  SEY6210.1, *MUP1-pHluorin::KanMX6*  SEY6210, *MUP1-pHluorin::natMX6*, *vps35Δ::KanMX6, snx4Δ::TRP1, mvp1Δ::hphNT1*  SEY6210, *pep4Δ::LEU2*, *prb1Δ::LEU2*, *vps55Δ::KanMX6*, *MVP1-GFP::TRP1*  SEY6210, *VPS5-3xFLAG::HIS3*  SEY6210, *VPS17-3xFLAG::HIS3*  SEY6210, *SNX4-3xFLAG::HIS3*  SEY6210, *SNX41-3xFLAG::HIS3*  SEY6210, *SNX42-3xFLAG::HIS3*, *SNX41-3xHA::TRP1*  SEY6210, *YKR078w-3xFLAG::HIS3*  SEY6210, *vps55Δ::KanMX6, VPH1-mCherry::TRP1*  SEY6210, *pep4Δ::LEU2*, *prb1Δ::LEU2*, *vps1Δ::hphNT1, MVP1-3xFLAG::HIS3*  SEY6210, *sec18ts, VPH1-mCherry::TRP1*  SEY6210, *sec18ts, VPH1-mCherry::TRP1, VPS55-3xFLAG::HIS3*  SEY6210, *sec18ts, VPH1-mCherry::TRP1, VPS55-GFP::LEU2*  SEY6210, *sec18ts, VPH1-mCherry::TRP1, VPS10-3xFLAG::HIS3, VPS55-GFP::LEU2*  SEY6210, *VPS17-3xHA::TRP1, VPS26-13xMyc::hphNT1*  SEY6210, *VPS5-3xFLAG::HIS3*, *VPS17-3xHA::TRP1, VPS26-13xMyc::hphNT1*  SEY6210, *VPS17-3xHA::TRP1*  SEY6210, *VPS17-3xHA::TRP1,MVP1-3xFLAG::HIS3*  SEY6210, *MVP1-GFP::HIS3,* *vam3Δ::LEU2*  SEY6210, *vps35Δ::KanMX6, snx4Δ::TRP1, mvp1Δ::hphNT1, MVP1-3xFLAG::URA3*  SEY6210, *vps35Δ::KanMX6, snx4Δ::TRP1, mvp1Δ::hphNT1, MVP1(I346E/Q468E/W496E)-3xFLAG::URA3* | (1)  (1)  This study  This study  This study  This study  This study  This study  This study  This study  This study  This study  This study  (2)  This study  This study  This study  This study  This study  This study  This study  This study  This study  (2)  This study  This study  This study  This study  This study  This study  This study  This study  This study  This study  This study  This study  This study  This study  This study  This study  This study  This study  This study  This study  This study  This study  This study  This study  This study  This study  This study  This study  This study  This study  This study  This study  This study  This study  This study  This study  This study  This study  This study  This study  This study  This study  This study  This study  This study  This study  This study  This study  This study  This study  This study  (2)  This study  This study  This study  This study |

**Supplemental File 1B. Plasmids used in this study**

| **Name** | **Genotype** | **Source** |
| --- | --- | --- |
| pRS305 [vec]  pRS416 [vec]  pRS426 [vec]  pRS416-VPS55-GFP  pRS416-mNeonGreen-PEP12  pRS416-MVP1-GFP  pRS416-MVP1(R172E)-GFP  pRS416-MVP1(I346E/Q468E/W496E)-GFP  pRS306-MVP1-FLAG  pRS306-MVP1(R172E)-FLAG  pRS306-MVP1(I346E/Q468E/W496E)-FLAG  pRS305-VPS55-GFP  pRS305-VPS55(60-63A)-GFP  pRS305-VPS55(64-67A)-GFP  pRS305-VPS55(68-71A)-GFP  pRS305-VPS55(72-75A)-GFP  pRS305-VPS55(133-136A)-GFP  pRS305-VPS55(137-140A)-GFP  pRS305-VPS55(K60A)-GFP  pRS305-VPS55(Y61A)-GFP  pRS305-VPS55(H62A)-GFP  pRS305-VPS55(T63A)-GFP  pRS305-VPS55(S64A)-GFP  pRS305-VPS55(D65A)-GFP  pRS305-VPS55(F66A)-GFP  pRS305-VPS55(M67A)-GFP  pRS305-VPS55(Y61A/T63A)-GFP  pRS305-VPS55(F66A/M67A)-GFP  pRS305-VPS55(Y61A/T63A/F66A/M67A)-GFP  pRS416-VPS55-FLAG  pRS416-VPS55(Y61A/T63A/F66A/M67A)-FLAG  pRS426-VPS55-GFP  pRS416-VPS1-GFP  pRS416-VPS1(K42A)-GFP  pRS416-VPS1(G476D)-GFP  pRS416-VPS1(K42A)-BFP  pRS305-VPS1-GFP  pRS416-GFP-VPS21  pRS416-KEX2-GFP  pRS416-GFP-NEO1  pRS416-NHX1-GFP  pRS426-NEO1  pRS416-MUP1-GFP  pRS416-GFP-CPS  pRS416-mCherry-ALP  pRS415-VPH1-mCherry  pRS416-GFP-FYVE  pRS425-VPS68  pEGFP-C2  pEGFP-SNX8  pET28a-Mvp1  pET28a-Vps1-GFP | *CEN URA3*  *2μ URA3*  *pRS416-VPS55pr-VPS55-GFP*  *pRS416-PEP12pr-mNeonGreen-PEP12*  *pRS416-MVP1pr-MVP1-GFP*  *pRS416-MVP1pr-MVP1(R172E)-GFP*  *pRS416-MVP1pr-MVP1(I346E/Q468E/W496E)-GFP*  *pRS306-MVP1pr-MVP1-3xFLAG*  *pRS306-MVP1pr-MVP1(R172E)-3xFLAG*  *pRS306-MVP1pr-MVP1(I346E/Q468E/W496E)-3xFLAG*  *pRS305-VPS55pr-VPS55-GFP*  *pRS305-VPS55pr-VPS55(60-63A)-GFP*  *pRS305-VPS55pr-VPS55(64-67A)-GFP*  *pRS305-VPS55pr-VPS55(68-71A)-GFP*  *pRS305-VPS55pr-VPS55(72-75A)-GFP*  *pRS305-VPS55pr-VPS55(133-136A)-GFP*  *pRS305-VPS55pr-VPS55(137-140A)-GFP*  *pRS305-VPS55pr-VPS55(K60A)-GFP*  *pRS305-VPS55pr-VPS55(Y61A)-GFP*  *pRS305-VPS55pr-VPS55(H62A)-GFP*  *pRS305-VPS55pr-VPS55(T63A)-GFP*  *pRS305-VPS55pr-VPS55(S64A)-GFP*  *pRS305-VPS55pr-VPS55(D65A)-GFP*  *pRS305-VPS55pr-VPS55(F66A)-GFP*  *pRS305-VPS55pr-VPS55(M67A)-GFP*  *pRS305-VPS55pr-VPS55(Y61A/T63A)-GFP*  *pRS305-VPS55pr-VPS55(F66A/M67A)-GFP*  *pRS305-VPS55pr-VPS55(Y61A/T63A/ F66A/M67A)-GFP*  *pRS416-VPS55pr-VPS55-3xFLAG*  *pRS416-VPS55pr-VPS55(Y61A/T63A/F66A/M67A)-3xFLAG*  *pRS426-VPS55pr-VPS55-GFP*  *pRS416-VPS1pr-VPS1-GFP*  *pRS416-VPS1pr-VPS1(K42A)-GFP*  *pRS416-VPS1pr-VPS1(G476D)-GFP*  *pRS416-VPS1pr-VPS1(K42A)-BFP*  *pRS305-VPS1pr-VPS1-GFP*  *pRS415-VPS21pr-GFP-VPS21*  *pRS416-KEX2pr-KEX2-GFP*  *pRS416-NEO1pr-GFP-NEO1*  *pRS416-NHX1pr-NHX1-GFP*  *pRS426-NEO1pr-NEO1*  *pRS416-MUP1pr-MUP1-GFP*  *pRS416-CPSpr-GFP-CPS*  *pRS416-CPYpr-mCherry-PHO8*  *pRS415-VPH1pr-VPH1-mCherry*  *pRS416-ADHpr-GFP-2xFYVE^EEA1^*  *pRS425-VPS68pr-VPS68*  *pCMVpr-EGFP*  *pCMVpr-EGFP-SNX8*  *pET28a-6xHis-SUMO-MVP1*  *pET28a-6xHis-SUMO-VPS1-GFP* | (3)  (3)  (3)  This study  This study  This study  This study  This study  This study  This study  This study  This study  This study  This study  This study  This study  This study  This study  This study  This study  This study  This study  This study  This study  This study  This study  This study  This study  This study  This study  This study  This study  This study  This study  This study  This study  This study  This study  This study  This study  This study  This study  This study  This study  This study  This study  This study  This study  Clontech, #632481  This study  This study  This study |

**Supplemental References**

1. Robinson, J.S., Klionsky, D.J., Banta, L.M., and Emr, S.D. (1988) Protein sorting in Saccharomyces cerevisiae: isolation of mutants defective in the delivery and processing of multiple vacuolar hydrolases. Mol. Cell Biol. *8*, 4936-4948.

2. Suzuki, S.W., Chuang, Y.S., Li, M., Seaman, M.N.J., and Emr, S.D. (2019). A bipartite sorting signal ensures specificity of retromer complex in membrane protein recycling. J. Cell Biol. *218(9)*, 2876-2886.

3. Sikorski, R.S., and Hieter, P. (1989) A system of shuttle vectors and yeast host strains designed for efficient manipulation of DNA in Saccharomyces cerevisiae. Genetics *122*, 19-27.
